# Choice of specimen’s extraction site affects wound morbidity in laparoscopic colorectal cancer surgery

**DOI:** 10.1007/s00423-022-02701-7

**Published:** 2022-10-11

**Authors:** Mahmood Al Dhaheri, Mohanad Ibrahim, Omer Al-Yahri, Ibrahim Amer, Mahwish Khawar, Noof Al-Naimi, Ayman Abdelhafiz Ahmed, Mohamed Abu Nada, Amjad Parvaiz

**Affiliations:** 1 Colorectal Surgery Unit, Hamad Medical Corporation, PO Box 3050, Doha, Qatar; 2 General Surgery, Hamad Medical Corporation, PO Box 3050, Doha, Qatar; 3 Champalimaud Foundation, Lisbon, Portugal

**Keywords:** Laparoscopic surgery, Colorectal cancer, Surgical site infection, Incisional hernia, Specimen extraction site

## Abstract

**Background:**

The choice for an ideal site of specimen extraction following laparoscopic colorectal surgery remains debatable. However, midline incision (MI) is usually employed for right and left–sided colonic resections while left iliac fossa or suprapubic transverse incision (STI) were reserved for sigmoid and rectal cancer resections.

**Objective:**

To compare the incidence of surgical site infection (SSI) and incisional hernia (IH) in elective laparoscopic colorectal surgery for cancer and specimen extraction via MI or STI.

**Method:**

Prospectively collected data of elective laparoscopic colorectal cancer resections between January 2017 and December 2019 were retrospectively reviewed. MI was employed for right and left–sided colonic resections while STI was used for sigmoid and rectal resections. SSI is defined according to the US CDC criteria. IH was diagnosed clinically and confirmed by CT scan at 1 year.

**Results:**

A total of 168 patients underwent elective laparoscopic colorectal resections. MI was used in 90 patients while 78 patients had STI as an extraction site. Demographic and preoperative data is similar for two groups. The rate of IH was 13.3% for MI and 0% in the STI **(*p* = 0.001)**. SSI was seen in 16.7% of MI vs 11.5% of STI (*p* = 0.34). Univariate and multivariate analysis showed that the choice of extraction site is associated with statistically significant higher incisional hernia rate.

**Conclusion:**

MI for specimen extraction is associated with higher incidence of both SSI and IH. The choice of incision for extraction site is an independent predicative factor for significantly higher IH and increased SSI rates.

## Introduction

Minimally invasive surgery (MIS) for colorectal surgery is associated with a number of widely recognized short-term benefits, which include accelerated postoperative recovery, shorter length of hospital stay and decreased postoperative pain [1]. However, the widely reported claims for reduced incidence of incisional hernias and surgical site infections associated with MIS approach when compared to open surgery remain controversial [2].

Following the completion of MIS procedure in colorectal surgery, an extraction incision at the abdominal wall is required to remove the specimen. The choice for the incision site of extraction remains at the discretion of the operating surgeon. In most cases, either the periumbilical midline or suprapubic transverse incision is deployed to extract the resected specimen. Both SSI and IH remain the major concern following the specimen extraction in laparoscopic colorectal surgery.

Although, the impact of specimen’s extraction site on both SSI and IH has been evaluated in the literature [3–8], however, none of the studies have directly compared midline incision (MI) and suprapubic transverse incision (STI) as an extraction site following elective laparoscopic colorectal cancer resection.

This study aims to evaluate the incidence of both IH and SSI comparing both midline and suprapubic transverse abdominal incision following laparoscopic colorectal resections for cancer, and the risk factors associated with both IH and SSI are also analyzed.

## Methods

Prospectively collected data for consecutive patients who underwent elective laparoscopic colorectal resection for primary cancer from January 2017 to December 2019 at a tertiary referral centre were retrospectively reviewed. Patients who required conversion to open surgery or had a complication that needed reoperation with open laparotomy were excluded from analysis.

All locally advanced rectal tumors, stage III and IV colon cancer were recommended to receive neoadjuvant chemoradiotherapy and adjuvant chemotherapy respectively. All eligible patients with resectable colorectal cancer were considered for MIS except for anesthesia contraindications, previous laparotomies, or those who needed multvisceral resection.

All patients received pre-operative prophylactic antibiotics at induction (cefuroxime 1.5 g, and metronidazole 500 mg) and for penicillin allergy (clindamycin 600 mg and gentamicin 80 mg).

The extraction-site incisions were classified into two groups: midline incision (MI) and suprapubic transverse incision (STI) (Pfannenstiel). Both MI and STI have median size of 6 cm (range of 5–10 cm) which were routinely measured and documented for every patient. The midline extraction wounds were either supraumbilical or periumbilical. Incisions were made using sharp division of the linea alba and were closed using a single layer large bite 1 cm from the edge and 1 cm apart using PDS loop no. 1 (Ethicon Inc., Cincinnati, Ohio, USA).

For STI, the incisions were made using sharp division of the anterior rectus sheath with blunt spreading of the muscular layers and were closed in two layers with no. 2/0 Vicryl to approximate the muscle and no. 1 Vicryl to close the sheath transversely. All procedures were completed with laparoscopic approach, and extraction site was only used to retrieve the resected specimen. MI was used for right and left colonic resections while STI was reserved for sigmoid and rectal cancer resections. All right-sided and left hemicolectomies anastomoses were performed extracorporeal while sigmoid and rectum were intracorporeal. A wound protector (Alexis Applied medical) was used in all cases during specimen’s extraction.

Data for age, gender, body mass index (BMI), diabetes, preoperative albumin and hemoglobin, neoadjuvant and adjuvant chemoradiotherapy, and post-operative complication including wound hematoma and anastomotic leakage were compared between the two groups were classified according to Clavien-Dindo classification [9].

### Definition of incisional hernia and surgical site infection

An IH is defined clinically as the presence of swelling and cough impulse along the extraction incision site, and this was confirmed by CT scan which performed routinely as part of surveillance for the cancer patients.

SSI was diagnosed according to definition set by the Centers for Disease Control and Prevention in 1992 [10].

### Surgical follow-up

Following discharge, patients were reviewed at 2 weeks, 3 months, and 6-monthly intervals for 5 years. A CT scan of abdomen, pelvis and chest was also performed annually for all patients.

### Statistical analysis

Chi-square or Fisher exact test was used to compare the categorical data and Mann–Whitney *U* test for the numerical data.

Finally, univariate binary logistic regression analysis was performed on patients receiving midline or suprapubic incision to assess whether the choice of incision (MI vs STI) affected morbidity (SSI and IH) and following this, a multivariate model was applied. The constant was included in the analysis model and data is presented as odds ratio, 95% confidence interval and *p* value.

Regression analysis was not possible for incisional hernias because there were no IH in the STI group. For the purpose of data analysis, one of the STI patients was randomly assigned an IH.

Statistical analysis for the data was performed using SPSS V26 IBM, New York, USA.

## Results

During the 3-year period, a total of 168 patients received elective laparoscopic colorectal resection for cancer. The baseline characteristics and demographics for all the patients are outlined in Table 1. There was no significant difference observed in the demographics between the two compared groups. Median follow-up for the patients was 29 months (IQR 12–44 months) MI was used for right and left hemicolectomy while STI was deployed only for sigmoid and rectal resections (Table 1).

**Table 1:** The demographic data and post-operative outcomes

Characteristic	Midline (90)	Suprapubic (78)	*p*-value
Age: Median (IQR)	54 (43–60)	55 (48–65)	0.08
Gender M/F %	66.7/ 33.3%	62.8%/37.2%	0.6
BMI Median (IQR)	27 (24–31)	27 (23–30)	0.56
Comorbidities			
DM	30%	26%	
HTN	36%	35%	
Previous surgery			0.2
Open abdominal surgery	16.7%	7.7%	
Laparoscopic surgery	15.6%	14.1%	
Smoking %	24%	16%	0.2
Location of tumor			< 0.001
Right sided tumor	70%	0	
Left sided tumor (splenic and descending colon)	30%	0	
Sigmoid colon and rectum	0	100%	
Neoadjuvant Radiotherapy	3.3%	16.%	0.04
Pre Op HGB Median (IQR)	11 (10–13)	12 (10–13)	0.07
Pre Op albumin Median (IQR)	36 (34–40)	37 (34–40)	0.29
Procedure			
Right hemicolectomy	68.8%	0	
Left hemicolectomy	30%	0	< 0.001
Anterior resection	0	84.6%	
Low anterior resection	0	15.4%	
Small bowel resection	1.1%		
Post Op complication			
Wound infection	16.7% (15)	11.5% (10)	0.34
Incisional hernia	13.3% (12)	0	< 0.001
Anastomotic leakage	0	3.8%	
Wound hematoma	1.1%	3.8%	
Anastomotic site bleeding	2.2%	1.3%	0.106
Intraabdominal infection	2.2%	0	
Stage I	26%	25.6%	
Stage II	30%	21.7%	
Stage III	37%	48.7%	0.04
Stage IV	7%	4%	
Adjuvant chemotherapy	46%	55.7%	0.04
Median follow-up	29 months	29 months	

*DM*, Diabetes mellitus; *HTN*, hypertension; *IQR*, inter-quartile range; *HGB*, hemoglobin; Number between two brackets ()

Midline incision (MI) was used in 90 (54%) of patients and suprapubic transverse incision (STI) in 78 (46%) of patients. In our study, the overall incidence of IH for all patients was 7% and SSI 14%.

MI was associated with significantly higher rate of IH (13.3%) when compared to STI (0%) (*P* = 0.001). Similarly, the rate of SSI was also higher in MI (16.7%) when compared to STI (11.5%) (*P* = 0.3) although the difference is not significant.

Other risk factors such as high BMI, pre-operative low hemoglobin and albumin were not seen as risk factors for development of IH and SSI in our study.

Univariate logistic regression analysis of all 168 cases showed no significant factor other than the choice of extraction site affected the morbidity (SSI and IH) (*P* = 0.019). This was still the case in multivariate analysis when other clinically relevant factors were adjusted for as detailed in (*P* = 0.018) Table 2.

**Table 2:** Univariate and multivariate analysis of the risk factors for IH and SSI

		Univariate analysis			OR	Multivariate analysis		
	OR	95% CI lower	95% CI upper	*p* value	OR	95% CI lower	95% CI upper	*p* value
Incision site (midline vs suprapubic)	11.846	1.504	93.331	0.019	15.868	1.619	155.523	0.018
Gender (male vs female)	2.311	0.739	7.228	0.150	2.815	0.721	10.994	0.136
Age	0.991	0.948	1.035	0.670	0.975	0.931	1.022	0.298
BMI	0.977	0.890	1.072	0.621	1.013	0.906	1.133	0.820
Smoking	0.867	0.225	3.337	0.836	1.023	0.199	5.258	0.978
Pre op HGB	1.286	0.156	10.587	0.401	0.965	0.627	1.485	0.870
Pre op albumin	1.070	0.958	1.194	0.229	1.067	0.931	1.223	0.350
SSI	0.522	0.133	2.055	0.353	0.717	0.148	3.471	0.679
Neoadjuvant chemotherapy	1.286	0.156	10.587	0.815	0.229	0.014	3.746	0.301
Adjuvant chemotherapy	2.527	0.747	8.555	0.136	2.022	0.520	7.871	0.310

*OR*, odds ratio; *BMI*, body mass index; *SSI*, surgical site infection

## Discussion

Previously, several variables have been identified as independent risk factors associated with IH and SSI related with wound extraction site following laparoscopic colorectal surgery.

It includes midline specimen extraction site, increased BMI, old age, female gender, malnutrition, anemia, chronic disease such as diabetes mellitus (DM) and chronic obstructive pulmonary disease (COPD) [2, 4, 6]. The present study reports on the incidence IH and SSI in the specific specimen extraction sites following laparoscopic colorectal resection for cancer patients and focus on the risk factors associated with such wound morbidity. We believe that this is the first study looking at the impact of extraction site in elective laparoscopic colorectal surgery for patients with colorectal cancer.

Wound morbidity is common following open or laparoscopic colorectal surgery with incidence of IH rates range from 5 to 15% and SSI (3–26%) with 10% incidence in elective laparoscopic colorectal resection [7, 8, 11–14].

Published evidence has shown that MI has higher IH rates [15–19]. In a study by Singh R et al. that looked at the impact of extraction site on the incidence of IH in laparoscopic colorectal surgery, found incidence of IH of 7.8% which were all associated with midline incision [4].

The published data indicate that the site selected for specimen extraction is both the most critical and, in some cases, the most easily modifiable variable. Although there is a difference in the mode of closure between MI and STI in terms of the suture material, the literature showed no significant impact of such difference [20].

In our study the incidence of IH was 7% which correlates well with the published figures in the literature, and the SSI rates of 14% which is slightly higher than reported incidence for most elective colorectal surgery.

Subgroup analysis for MI and STI showed that IH was only present in MI 13.3% and none in the STI. This result is both clinically and statistically significant *P* = 0.001. SSI was also higher in the MI group 16.7% vs 11.5% in STI *P* = 0.3. The findings indicate that the choice of the midline extraction site was associated with statistically significant higher incisional hernia rates, and increased SSI rates although not significant. Univariate and multivariate analysis showed that the choice of incision site is an independent predictive factor for IH but failed to show significant difference between the groups for the development of SSI.

Although development of incisional hernia is related to length of follow-up, we believe that the median follow-up 29 months in our study is a reasonable measure of this outcome.

The slightly higher rate of SSI in our study may be related to the absence of mechanical bowel preparation with oral antibiotic which has proven to minimize the risk of SSI in some studies [21–24].

This present study also suffers from some limitations. The small sample size and retrospective nature of the review may pose some bias; however, the homogeneity of patients’ pathology, comparative demographics and intensive clinical follow-up with the use of CT scan to aid the diagnosis of IH provide some strength to our data too.

We found that the MI is mainly employed to extract specimen following right and left colectomy. This may be a surgeon’s choice due to limited mobility of the colon making it a preferred choice to extract specimen and perform extracorporeal anastomosis as well. We believe and propose that MI incision should be avoided as extraction site and should be replaced by STI. It would need expertise in performing of intracorporeal anastomosis technique.

## Conclusion

The choice of incision for extraction site is an independent predicative factor for significantly higher IH and increased SSI rates.

Suprapubic transverse incision should be the preferred choice for a specimen extraction following laparoscopoic colorectal surgery. MI for specimen extraction should be avoided due to higher incidence of IH.

## References

[1] Weeks JC, Nelson H, Gelber S, Sargent D (2011). vs Open colectomy for colon cancer. Surg.

[2] Klaristenfeld DD, McLemore EC, Li BH (2015). Significant reduction in the incidence of small bowel obstruction and ventral hernia after laparoscopic compared to open segmental colorectal resection. Langenbecks Arch Surg.

[3] Samia H, Lawrence J, Nobel T (2013). Extraction site location and incisional hernias after laparoscopic colorectal surgery: should we be avoiding the midline?. Am J Surg.

[4] Singh R, Omiccioli A, Hegge S, McKinley C (2008). Does the extraction-site location in laparoscopic colorectal surgery have an impact on incisional hernia rates?. Surg Endosc Interv Tech.

[5] Orcutt ST, Balentine CJ, Marshall CL (2012). Use of a Pfannenstiel incision in minimally invasive colorectal cancer surgery is associated with a lower risk of wound complications. Tech Coloproctol.

[6] DeSouza A, Domajnko B, Park J (2011). Incisional hernia, midline versus low transverse incision: what is the ideal incision for specimen extraction and hand-assisted laparoscopy?. Surg Endosc.

[7] Cm S, A D, MK D,  (2009). Midline versus transverse incision in major abdominal surgery: a randomized, double-blind equivalence trial (POVATI: ISRCTN60734227). Ann Surg.

[8] Ku DH, Kim HS, Shin JY (2020). Short-term and medium-term outcomes of low midline and low transverse incisions in laparoscopic rectal cancer surgery. Ann Coloproctol.

[9] Dindo D, Demartines N, Clavien PA (2004). Classification of surgical complications: a new proposal with evaluation in a cohort of 6336 patients and results of a survey. Ann Surg.

[10] Horan TC, Gaynes RP, Martone WJ (1992). CDC definitions of nosocomial surgical site infections, 1992: a modification of CDC definitions of surgical wound infections. AJIC Am J Infect Control.

[11] Laurent C, Leblanc F, Bretagnol F (2008). Long-term wound advantages of the laparoscopic approach in rectal cancer. Br J Surg.

[12] Blumetti J, Luu M, Sarosi G (2007). Surgical site infections after colorectal surgery: do risk factors vary depending on the type of infection considered?. Surg.

[13] Tang R, Chen HH, Wang YL (2001). Risk factors for surgical site infection after elective resection of the colon and rectum: a single-center prospective study of 2,809 consecutive patients. Ann Surg.

[14] Poon JT, Law WL, Wong IW (2009). Impact of laparoscopic colorectal resection on surgical site infection. Ann Surg.

[15] Lee L, Abou-Khalil M, Liberman S (2017). Incidence of incisional hernia in the specimen extraction site for laparoscopic colorectal surgery: systematic review and meta-analysis. Surg Endosc.

[16] Lee L, Mata J, Droeser RA (2018). Incisional hernia after midline versus transverse specimen extraction incision. Ann Surg.

[17] Varathan N, Rotigliano N, Nocera F (2020). Left lower transverse incision versus Pfannenstiel-Kerr incision for specimen extraction in laparoscopic sigmoidectomy: a match pair analysis. Int J Colorectal Dis.

[18] Benlice C, Stocchi L, Costedio MM (2016). Impact of the specific extraction-site location on the risk of incisional hernia after laparoscopic colorectal resection. Dis Colon Rectum.

[19] Griffith KC, Clark NV, Mushinski AA (2018). Incisional outcomes of umbilical vs suprapubic mini-laparotomy for tissue extraction: a retrospective cohort study. J Minim Invasive Gynecol.

[20] Na H, Eb D, L V,  (2018). Meta-analysis on materials and techniques for laparotomy closure: the MATCH review. World J Surg.

[21] Ohman KA, Wan L, Guthrie T (2017). Combination of oral antibiotics and mechanical bowel preparation reduces surgical site infection in colorectal surgery. J Am Coll Surg.

[22] Cannon JA, Altom LK, Deierhoi RJ (2012). Preoperative oral antibiotics reduce surgical site infection following elective colorectal resections. Dis Colon Rectum.

[23] Kiran RP, Murray ACA, Chiuzan C (2015). Combined preoperative mechanical bowel preparation with oral antibiotics significantly reduces surgical site infection, anastomotic leak, and ileus after colorectal surgery. Ann Surg.

[24] Vadhwana B, Pouzi A, Surjus Kaneta G (2020). Preoperative oral antibiotic bowel preparation in elective resectional colorectal surgery reduces rates of surgical site infections: a single-centre experience with a cost-effectiveness analysis. Ann R Coll Surg Engl.

